# PET-scan in diagnosis of non-bacterial thrombotic endocarditis: a case report

**DOI:** 10.1186/s43044-024-00452-y

**Published:** 2024-02-13

**Authors:** Konstantinos Papakonstantinou, Filippos-Paschalis Rorris, Nikoleta Stanitsa, John Kokotsakis

**Affiliations:** Cardiovascular and Thoracic Surgery Department, Evaggelismos General Hospital, 45-47 Ypsilantou Street, 10676 Athens, Greece

**Keywords:** Adenocarcinoma, Endocarditis, Non-infective, Thrombosis, Neoplasms

## Abstract

**Background:**

Non-bacterial thrombotic endocarditis (NBTE), also known as marantic endocarditis or Libman–Sacks endocarditis, is a rare non-infectious condition affecting mostly the left-sided heart valves of patients who, most often, suffer from malignancies and connective tissue disorders. Herein, we present a case of a male patient with marantic endocarditis due to occult lung adenocarcinoma.

**Case presentation:**

The patient fulfilled the modified Duke’s criteria of possible bacterial endocarditis of the aortic valve; however, multiple blood cultures and serological tests were negative. In addition, the patient’s clinical course was constantly deteriorating. Thus, a fluorodeoxyglucose-positron emission tomography (FDG-PET) scan was conducted. This examination revealed multiple positive thoracic lymph node groups, as well as a positive lung lesion. The patient underwent aortic valve replacement and lymph biopsies during the operation established the diagnosis of solid/micropapillary lung adenocarcinoma and consequently of the non-bacterial thrombotic endocarditis.

**Conclusions:**

Advanced imaging techniques may be needed to diagnose NBTE and should be kept in mind when the Duke criteria are not definite. Clinical suspicion is key to implement these premises. However, the exact role of the PET-scan has yet to be specified.

## Background

Non-bacterial thrombotic endocarditis (NBTE), also known as marantic endocarditis or Libman–Sacks endocarditis, is a rare condition affecting individuals with malignant diseases, connective tissue disorders, hypercoagulopathy states, severe burns, and other chronic conditions [1, 2]. The prevalence of the disease is largely unknown, varying between 0.9 and 1.6% in autopsy series [2], and affecting almost 4% of all cancer patients [3]. Concerning the latter, it may be associated with various tumors as well as hematologic malignancies [2, 4, 5]. The aortic and mitral valves are most commonly affected. Although not so common, marantic endocarditis in prosthetic valves has also been documented, affecting the leaflets in a bioprosthesis, or the sewing ring in a mechanical heart valve [2, 6–8].

The condition is hallmarked by systemic embolization of aseptic, friable vegetations which are histologically unique consisting of fibrin, thrombi, and immune complex deposits [2, 5, 9]. In addition, most cases of marantic endocarditis reflect an advanced stage of cancer burden [4]. Herein, we present a case of a male patient diagnosed with marantic endocarditis and embolic phenomena due to occult lung adenocarcinoma, using the PET-scan.

This case report was prepared following the CARE guidelines [10].

## Case presentation

A 60-year-old, hypertensive, heavy smoker, male presented in the emergency department (ER) with chest discomfort, bloody sputum, mild fever, and a swollen right lower limb. Α duplex scan of the limb performed initially was positive for deep vein thrombosis. Further work-up with ventilation–perfusion lung scintigraphy (VQ) confirmed the diagnosis of pulmonary embolism. As a result, the patient was treated with an anticoagulation regimen, with gradual symptom remission. Furthermore, a chest computed tomography (CT) scan was carried out, revealing a 15-mm nodule at the lingula and a few mildly swollen para-aortic lymph nodes. The transthoracic echocardiography (TTE) performed at that time only showed a mild aortic insufficiency. Therefore, the patient was discharged home with instructions to perform a new scan for follow-up of the lung nodule and an upper and lower gastrointestinal endoscopy, as well as to undertake a thrombophilia test.

A month later, he presented again in the emergency department, with general weakness and gait instability. A brain CT scan revealed a recent left temporoparietal infarct. High values of hs-Troponin-I and inflammatory markers were also noted in his laboratory tests. Another TTE was performed, and a perforated coronary cusp was noted, thus suggesting the diagnosis of endocarditis. The finding was further confirmed with transesophageal echocardiography (TEE). The coronary angiogram showed marginal lesions in the right coronary artery.

**Fig. 1:**
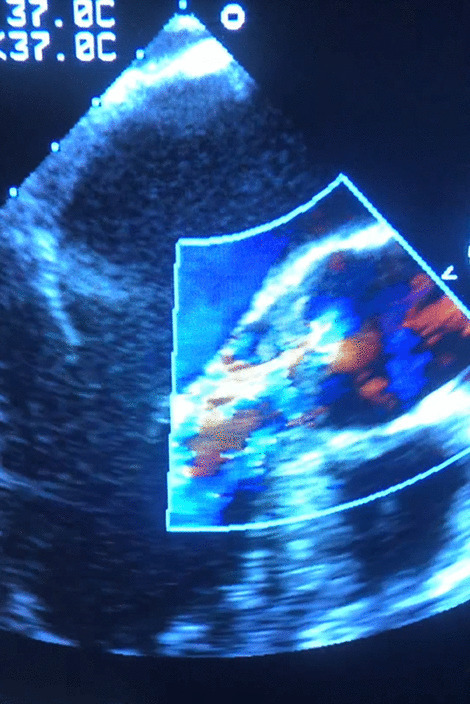
Aortic valve regurgitant jets seen in echocardiography

Few days later, a severe chest pain episode occurred with ST-segment elevations in the ECG, sharp rise of the Troponin levels, and anterior wall and apex motion abnormalities (WMA). A TTE finally revealed small vegetations in the aortic valve along with mild aortic insufficiency, in addition to the sustained apex WMA and regurgitation jets (Fig. 1). The diagnosis of infective endocarditis with embolic events in the brain and the coronary arteries was highly considered.

Multiple blood cultures, viral, immunological, and tumor marker tests (Roche SeptiFast® MGRADE PCR, Coxiella IgM and IgG antibodies, Widal-Wright test, lupus anticoagulant levels, b2-gpi antibodies, galactomannan antigen test, serum complement C3-C4, β-d-Glucan Test, Wright-Coombs test, and Coxsackie B antibodies) were all negative. Despite empirical antibiotic therapies, there was no remarkable clinical or serological change of state. In contrast, the patient’s condition deteriorated with fever, moderate renal failure, elevating inflammatory markers, and lower limb digit emboli. Additionally, a new CT scan revealed splenic infarcts. The patient fulfilled the modified Duke criteria for possible endocarditis (one major criterion—vegetations in the aortic valve) and two minor ones (fever and vascular phenomena) and was transferred to the Department of Cardiothoracic Surgery for urgent surgical management.

**Fig. 2:**
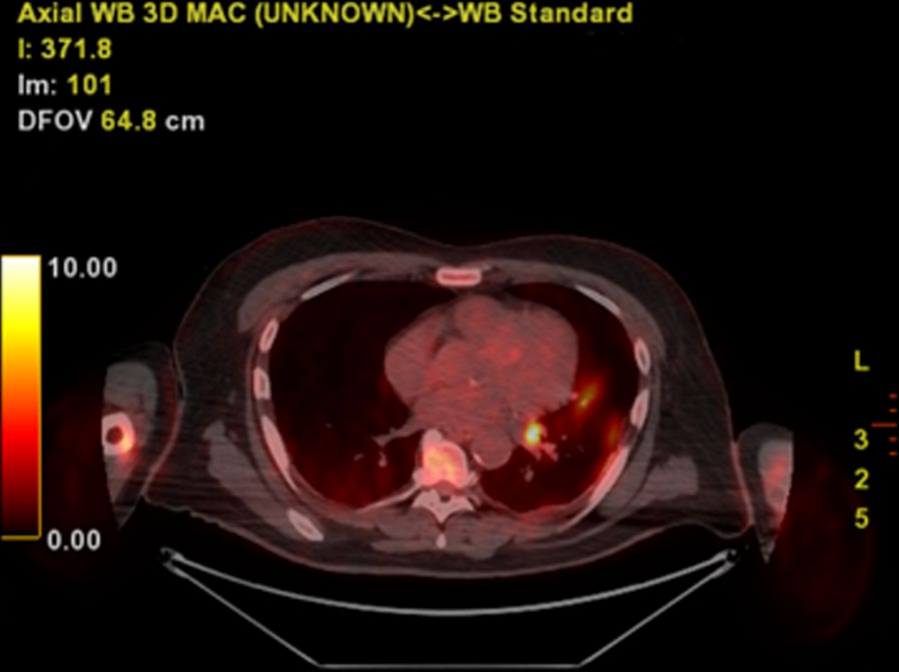
PET-CT scan showing no FDG uptake at the aortic valve region

**Fig. 3:**
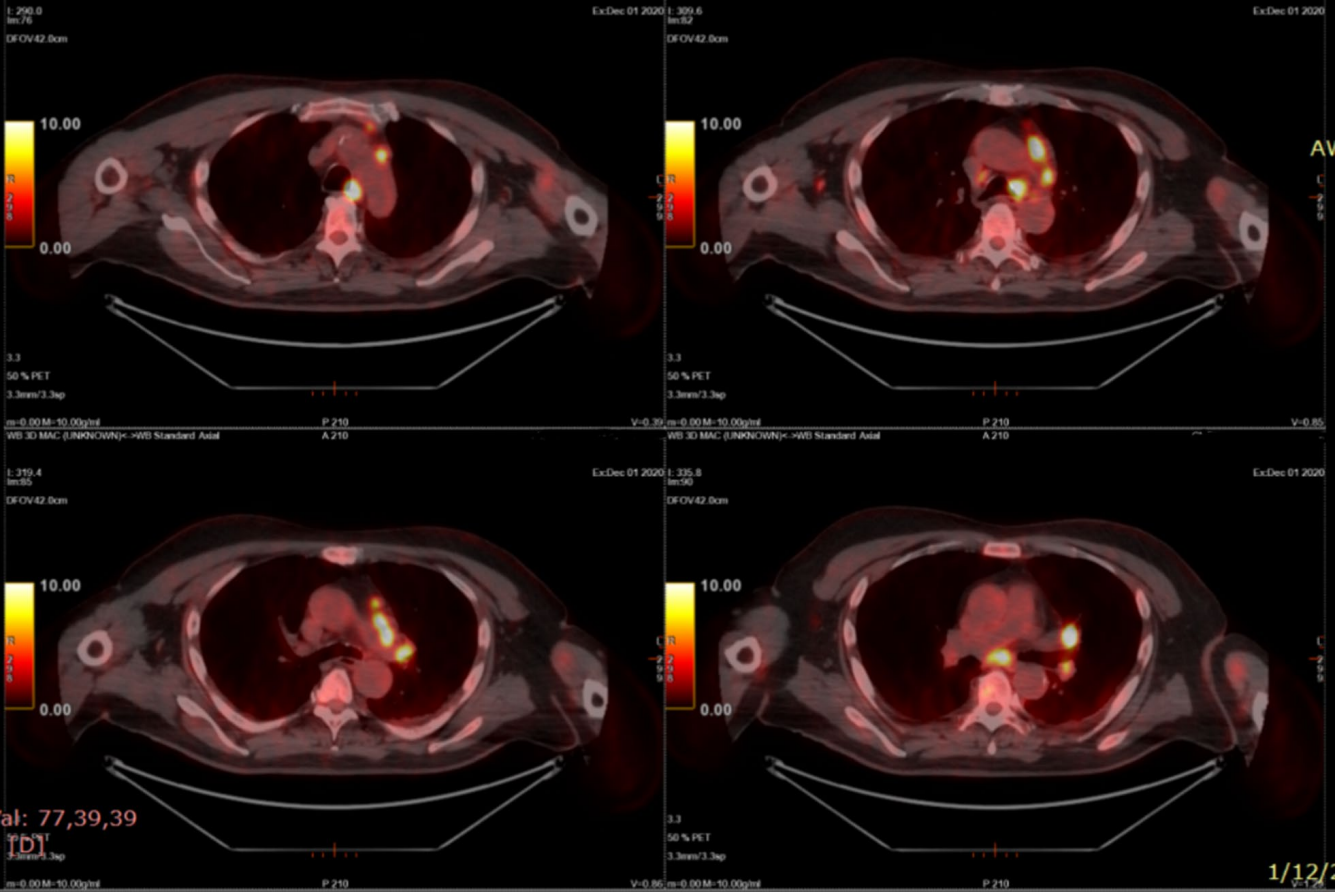
PET-CT scan showing increased uptake of FDG in multiple thoracic lymph node groups (prevascular anterior to the major vessels in upper mediastinum, para-aortic, left and right paratracheal, aorto-pulmonary window, subcarinal, and right and left hilar—SUVmax: 23,4)

**Fig. 4:**
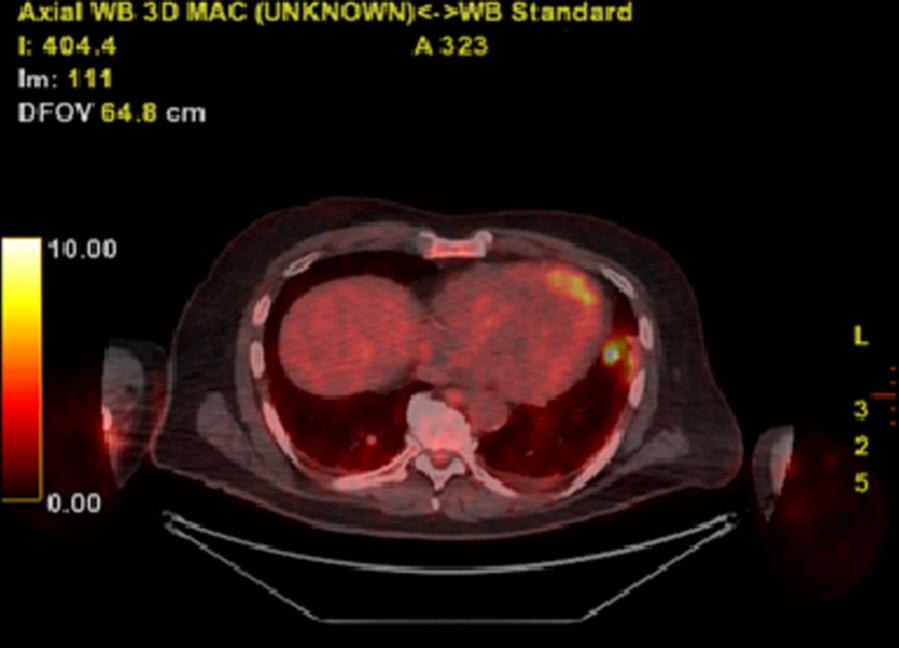
PET-CT scan showing increased FDG uptake from the lingula lesion (SUVmax: 13)

The rapid clinical deterioration and the multiple negative blood cultures despite a broad-spectrum antibiotic treatment posed a diagnostic dilemma. As a result, a fluorodeoxyglucose-positron emission tomography (FDG-PET) scan was conducted. The findings were conclusive: increased FDG uptake in numerous thoracic lymph node groups and the known lingula lesion but no uptake in the aortic valve (Figs. 2, 3, and 4).

### Treatment

After heart team consultation, the option of surgery was preferred for the replacement of the embologenic aortic valve and the biopsy of the mediastinal lymph nodes. So, an aortic valve replacement with a mechanical prosthesis and a resection of a para-aortic lymph node block was performed.

### Outcome

The native aortic valve had mild calcifications, no cusp destruction, and broad base small vegetations. The culture of the valve was negative, but the histological result of the lymph node block was solid/micropapillary lung adenocarcinoma. After this result, the diagnosis of marantic, non-thrombotic endocarditis was established.

A stormy postoperative course followed, including renal failure necessitating renal replacement therapy, and multiple brain infarcts due to disseminated intravascular coagulopathy, all of which led to the patient’s death 10 days postoperatively.

## Discussion

The term “marantic” stems from the Greek word—marantikos, which means wasting away, to emphasize the relation to wasting states, like cancer. This was the key to this case. Yet, raising clinical suspicion for the disease remains a challenge.

Embolization of friable vegetations is considered the main manifestation of NBTE [5], as was the case for our patient. However, echocardiography may not be sensitive enough to detect small particles of the vegetation (< 3 mm) that often remain attached to the valve after embolization [1]. This observation should alert clinicians to use an alternative imaging technique, when endocarditis is suspected [11].

Although vegetations were eventually found and despite broadened antibiotic treatment, the patient never accomplished disease recess. The modified Duke criteria were not met and based on existing guidelines [1], their sensitivity may be improved by new imaging techniques. This is what led us to obtain the PET-scan, which unveiled a possible malignancy and directed our decision-making process. Indeed, the “bacterial endocarditis” scenario seemed less likely, since there was no metabolic activity in the aortic valve position indicated by this imaging test. However, caution is advised when interpreting PET-scan results, as differential diagnosis should include multiple pathological conditions with similar FDG uptake pattern (active thrombi, soft atherosclerotic plaques, vasculitis, primary cardiac tumors, cardiac metastasis from a non-cardiac tumor, post-surgical inflammation, or foreign body reactions) [1].

The usefulness of the nuclear imaging also lies on the fact of suspecting the cancer diagnosis, through determining the presence or absence of extracardiac activity [12]. NBTE, in terms of cancer, is mostly associated with adenocarcinomas [5, 13] and constitutes a part of the known Trousseau syndrome [14], along with the venous involvement also seen in our case.

Due to the nature of the disease, data concerning the exact value of PET-scan on diagnosis of NBTE are scarce [12, 15]. As FDG PET-scan measures glucose metabolism, an abnormal radiotracer uptake has been observed before documented appearance of infectious damage in echocardiographic studies of infective endocarditis patients (11). Consequently, as cancerous cells exhibit high metabolic activity, an implication for early cancer detection and valve infiltration in NBTE patients may be formulated.

## Conclusions

PET-scan is an accessory imaging technique in non-bacterial thrombotic endocarditis diagnosis. The method, at present, is used when clinical suspicion for endocarditis is high, and other medical tests yield negative results. This imaging technique aids in practitioners’ decision-making process and in diagnostic dilemmas. However, more evidence is needed to specify the exact role of PET-scan in diagnosing non-bacterial thrombotic endocarditis.

## Data Availability

All data generated or analyzed during this study are included in this published article [and its supplementary information files].

## Abbreviations

NBTE: Non-bacterial thrombotic endocarditis
FDG-PET: Fluorodeoxyglucose-positron emission tomography
ER: Emergency department
VQ: Ventilation–perfusion lung scintigraphy
CT: Computed tomography
TTE: Transthoracic echocardiography
TEE: Transesophageal echocardiography
WMA: Wall and apex motion abnormalities
